# Sinonasal Mucosal Epithelioid Melanoma with Rapid Skull-Base and Orbital Progression

**DOI:** 10.3390/jcm15052068

**Published:** 2026-03-09

**Authors:** Vita Konopecka, Mārtiņš Blumbergs, Ingus Vilks, Gunta Seglina, Karina Biserova, Edgars Edelmers

**Affiliations:** 1 Bauska Hospital, Darza Street 7/1, LV-3901 Bauska, Latvia; vita.konopecka@rsu.lv; 2 Department of Otorhinolaryngology, Riga Stradins University, Pilsonu Street 13, LV-1002 Riga, Latvia; ingus.vilks@rsu.lv (I.V.); gunta.seglina@rsu.lv (G.S.); 3 Pauls Stradins Clinical University Hospital, Pilsonu Street 13, LV-1002 Riga, Latvia; martins.blumbergs@stradini.lv; 4 Department of Pathology, Riga Stradins University, 16 Dzirciema Street, LV-1007 Riga, Latvia; karina.biserova@rsu.lv; 5 Institute of Pathology, Pauls Stradins Clinical University Hospital, Pilsonu Street 13, LV-1002 Riga, Latvia; 6 Medical Education Technology Centre, Riga Stradins University, 26a Aninmuizas Boulevard, LV-1067 Riga, Latvia

**Keywords:** sinonasal mucosal epithelioid melanoma, skull base invasion, orbital extension

## Abstract

**Background**: Sinonasal mucosal melanoma is a rare and aggressive malignancy arising from the nasal cavity and paranasal sinuses, characterized by high local recurrence rates and poor survival. Skull-base and orbital progression can occur rapidly, particularly when preoperative imaging underestimates local extension. This paper reports a case of sinonasal mucosal epithelioid melanoma with fulminant postoperative skull-base breach and orbital invasion, highlighting its clinical course, management challenges, and histopathological features. **Methods**: A 60-year-old woman with progressive unilateral nasal obstruction, recurrent epistaxis, and headache underwent clinical evaluation, contrast-enhanced head MRI, CT, and PET-CT staging. Preoperative imaging demonstrated no intracranial or orbital invasion. Biopsy confirmed mucosal epithelioid melanoma with high proliferative activity (Ki-67 ~80–85%). The patient underwent extensive image-guided endoscopic resection with intraoperative cerebrospinal fluid leak repair. **Results**: Definitive histopathology confirmed pigmented epithelioid melanoma with extensive necrosis, bone invasion, and non-assessable resection margins due to specimen fragmentation (pT4a, Rx). Within two weeks postoperatively, CT and MRI demonstrated extensive local recurrence with cribriform plate destruction, anterior skull-base dural infiltration, and rapid orbital progression with optic nerve compression and loss of vision. Despite hemorrhage control and hypofractionated palliative radiotherapy (VMAT, 33 Gy in 11 fractions), the patient experienced progressive neurological decline, refractory pain, and recurrent tumour bleeding, and died approximately 4.5 months after initial presentation. **Conclusions**: In patients with sinonasal mucosal epithelioid melanoma, fulminant local progression with skull-base and orbital involvement may occur despite apparently limited preoperative imaging. When rapid vision loss, dural infiltration, and refractory nasal bleeding develop, structured palliation, hemorrhage control, and aggressive multimodal analgesia should be prioritized early alongside ongoing multidisciplinary decision-making.

## 1. Introduction

Sinonasal mucosal melanoma (SNMM) is an uncommon subtype of melanoma arising from melanocytes in the mucosa of the nasal cavity and paranasal sinuses. Despite representing a small fraction of all melanomas, SNMM is clinically important because it is frequently diagnosed at an advanced local stage and is associated with high rates of recurrence and poor disease-specific survival, even when apparently localized at presentation. Contemporary syntheses continue to report a guarded prognosis, with 5-year overall survival commonly in the range of ~25–35%, and local relapse remaining a dominant clinical problem [1,2]. The disease course is shaped by a combination of biological aggressiveness and the constraints of sinonasal anatomy, where early lesions may remain clinically silent until they are bulky or ulcerated and where complete resection with reliably assessable margins can be difficult to achieve [2,3,4].

Clinical presentation is typically nonspecific-nasal obstruction, recurrent epistaxis, rhinorrhea, facial pressure, and headache, contributing to diagnostic delay. Ophthalmic manifestations (diplopia, proptosis, epiphora, reduced visual acuity) and cranial neuropathies are generally late features, reflecting orbital or skull-base extension and heralding a rapid deterioration in quality of life and functional status [5]. Recent case-based literature continues to emphasize that ocular symptoms may be a prominent mode of presentation or a major complication in advanced sinonasal mucosal melanoma, underscoring the need for early recognition and coordinated ENT–ophthalmology–radiology assessment [5].

Accurate assessment of skull-base and orbital involvement is central to staging and treatment planning. Contemporary sinonasal malignancy guidance highlights multidisciplinary evaluation and careful delineation of local extension because skull-base and orbital invasion substantially alter management options and prognosis [6]. Contrast-enhanced MRI is generally preferred for soft-tissue assessment; however, systematic evidence indicates only moderate-to-high diagnostic performance for orbital and intracranial invasion across sinonasal malignancies, with clinically relevant false-negative and false-positive findings persisting despite modern imaging protocols [7]. These limitations are particularly consequential in tumours prone to multifocal submucosal spread or where postoperative inflammatory change may obscure early progression, creating a context in which clinicoradiologic–pathologic discordance can occur.

Surgery remains the cornerstone of management for localized SNMM, most commonly using endoscopic approaches in appropriate candidates, often combined with adjuvant radiotherapy to improve local control [8]. Achieving clear margins is repeatedly associated with improved outcomes, yet is challenging in practice due to complex anatomy, proximity to critical structures, and the propensity for discontinuous or submucosal tumour spread. In addition, endoscopic resections may be performed in a piecemeal fashion for technical reasons, which can complicate definitive margin assessment and introduce uncertainty into pathologic staging and postoperative risk stratification. Methods to improve specimen orientation and histosurgical mapping have been proposed to reduce ambiguity after endoscopic resections of sinonasal tumours and to strengthen the alignment between surgical intent and pathological reporting [9].

Management becomes particularly difficult when the tumour approaches or involves the orbit and anterior skull base. While frank intraconal invasion may necessitate aggressive measures, contemporary evidence supports orbit-preserving strategies in selected patients with sinonasal malignancies and orbital involvement, demonstrating oncologic outcomes comparable to orbit-sacrificing approaches while maintaining the possibility of a functionally useful eye [10]. This is consistent with the current multidisciplinary decision-making tendency that prioritizes individualized assessment of the degree of orbital invasion, expected morbidity, and patient-centred goals of care.

Radiotherapy is frequently used postoperatively or palliatively to improve local control and reduce morbidity from uncontrolled local disease, although survival benefit remains inconsistent across series and is influenced by stage, margin status, and the predominance of distant failure [8]. Modern techniques (IMRT/VMAT) and, in selected settings, charged particle therapy (e.g., protons) may improve target coverage near critical structures and can yield favourable early toxicity profiles; recent institutional experience specifically in sinonasal head-and-neck mucosal melanoma supports the feasibility of adjuvant proton therapy within multimodality regimens [11]. Systemic therapy has expanded in the era of immune checkpoint inhibition and targeted therapy, yet responses in mucosal melanoma are generally less frequent than in cutaneous melanoma, reflecting distinct molecular drivers and a comparatively low point-mutation burden with prominent structural variation [12]. Systematic syntheses in sinonasal mucosal melanoma highlight heterogeneous outcomes with immunotherapy and targeted approaches, with actionable alterations (e.g., KIT) present in a subset of patients, but with distant metastasis remaining a major determinant of survival [12].

Against this background, well-characterized case reports remain clinically valuable, particularly when they illustrate rapid postoperative progression, skull-base and orbital extension, or marked radiology-pathology discordance that alters trajectory toward symptom-directed care. Here, we report a fulminant course of sinonasal mucosal epithelioid melanoma with rapid postoperative skull-base breach and orbital progression, causing optic nerve compression and vision loss, despite initially negative preoperative intracranial/orbital imaging. The case emphasizes the need for early, protocolised palliation alongside multidisciplinary oncologic decision-making when skull-base invasion and vision-threatening orbital involvement emerge.

## 2. Case Presentation

This case report was prepared in accordance with the Declaration of Helsinki. Ethical approval was granted by the Ethics Committee of Riga Stradins University (protocol code 2-PĒK-4/581/2025, 31 March 2025).

A 60-year-old woman presented (13 February) with 3–4 months of progressive left-sided nasal obstruction, recurrent epistaxis, and unilateral headaches. She also reported intermittent left aural fullness; diplopia and visual impairment were initially denied. Prior head CT (24 January) showed a mass in the left nasal cavity with extension to the nasopharynx and right ethmoid cells. Past history included smoking, coronary artery disease with prior LAD PCI, hypercholesterolaemia, and chronic spinal pain. Baseline ECOG performance status was 0–1.

Clinical evaluation included otorhinolaryngological examination and biopsy. Imaging assessment comprised contrast-enhanced head MRI, CT of the paranasal sinuses with stereotactic planning, PET-CT, and postoperative CT and MRI of the head to evaluate local recurrence and skull-base/orbital extension.

A biopsy of an irregular, destructive polypoid lesion (13 February) was performed. Staging CT of chest and abdomen (28 February) revealed no abnormalities. Immunohistochemistry (10 March) showed Melan-A and HMB-45 positivity with high proliferation (Ki-67 ~80–85%); S-100 and epithelial/neuroendocrine markers were negative, supporting mucosal epithelioid melanoma with extensive tumour necrosis.

To perform morphological analysis, formalin paraffin-embedded tumour tissues were cut and stained with hematoxylin and eosin. For immunohistochemical analysis, Autostainer Link 48, staining with Daco antibodies for Ki-67, Melan-A, HMB-45, p40, p16, CD56, Synaptophysin, CD3, CD20, CD30, Actin, CD34, and CD99 were used. Histopathological examination was done by a pathologist using a light microscope (Nikon Eclipse Ci-L) at ×40 magnification.

**Figure 1:**
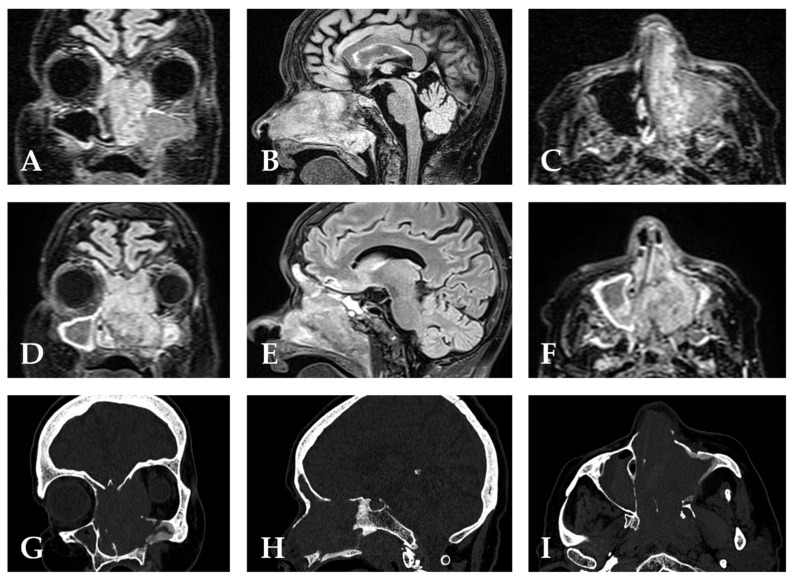
Pre-operative and post-operative neuroimaging in sinonasal mucosal epithelioid melanoma with rapid skull-base and orbital progression. **Pre-operative MRI** (**A**–**C**): (**A**) Coronal MRI demonstrating the primary left sinonasal mass with extension toward the medial orbital wall and ethmoid complex, without definitive periorbital soft-tissue invasion at this stage. (**B**) Sagittal 3D T2 SPACE DARK-FLUID (fluid-suppressed T2-weighted) MRI delineating the craniocaudal tumour extent, its relationship to the anterior skull base and nasopharynx, and absence of dural signal abnormality or intracranial involvement at this timepoint. (**C**) Axial MRI at the level of the nasal cavity and orbits; the arrow indicates the tumour abutting the left lamina papyracea and nasolacrimal duct/lacrimal sac region, without definitive orbital invasion on pre-operative imaging. **Post-operative MRI** (**D**–**F**): (**D**) Coronal post-operative MRI demonstrating rapid interval progression with increasing left orbital disease, extra-conal extension, extraocular muscle involvement, and progressive exophthalmos eleven days after extensive image-guided endoscopic resection. (**E**) Sagittal 3D T2 SPACE DARK-FLUID MRI revealing cribriform plate and olfactory cleft destruction with abnormal dural thickening and infiltration of the anterior cranial fossa floor—the fluid suppression highlighting tumour signal against suppressed CSF, consistent with radiologic upstaging to T4b behaviour. (**F**) Axial post-operative MRI confirming optic nerve compression at the canal and apex, pronounced exophthalmos, and extensive paranasal sinus and nasopharyngeal recurrence. **Post-operative CT** (**G**–**I**): (**G**) Coronal CT in bone window demonstrating extensive recurrent disease with destruction of the left lamina papyracea, ethmoid roof erosion, and progressive exophthalmos. (**H**) Sagittal CT illustrating the extent of anterior skull-base bony destruction and the surgical defect following Draft III frontal sinusotomy and bilateral sphenoidotomies. (**I**) Axial CT confirming diffuse paranasal sinus and nasopharyngeal involvement with post-surgical changes; no intracranial mass effect was identified on CT despite MRI-evident dural infiltration on concurrent T2 SPACE DARK-FLUID imaging.

Contrast-enhanced head MRI (10 March) showed no intracranial mass or orbital invasion. Multidisciplinary tumour board (18 March) recommended surgery and PET/CT. Stereotactic CT of the paranasal sinuses (25 March) demonstrated a large left nasal cavity mass extending to the nasopharynx and right ethmoid cells, with bony destruction of the left lamina papyracea and medial maxillary wall (as depicted in Figure 1), involvement of the nasolacrimal duct/lacrimal sac, and largely opacified frontal sinuses. PET-CT (27 March) showed a hypermetabolic primary in the nasal cavity without distant uptake.

On 28 March, the patient underwent extensive image-guided endoscopic tumour resection. The main mass involved the middle turbinate and cranial septum; per endoscopy, there was no periorbital soft-tissue invasion. Procedures included right infundibulotomy, left type III antrostomy, bilateral sphenoidotomies, bilateral middle turbinectomies, frontal sinusotomy (Draft III), and intraoperative CSF leak repair. Estimated blood loss was ~650 mL; a postoperative packed red-cell transfusion was given. She was discharged on 12 April with routine postoperative care and surveillance.

**Figure 2:**
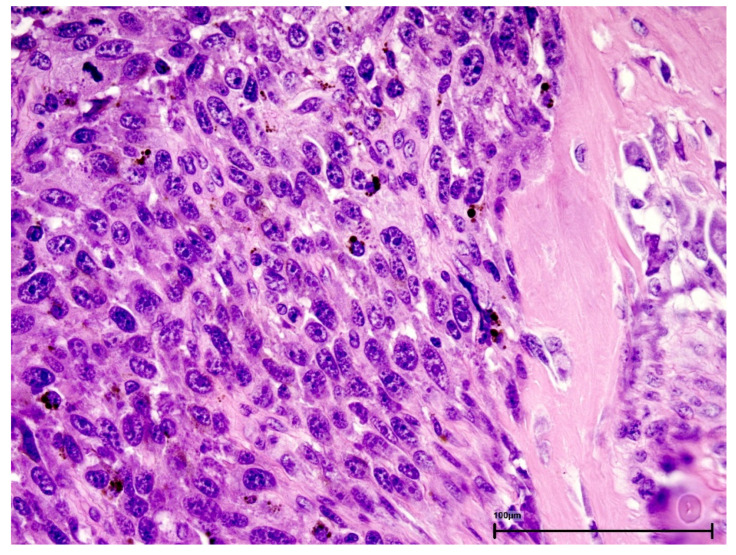
Pigmented nodular epithelioid cell melanoma invading bone tissues (hematoxylin and eosin, ×40).

Definitive histology reported pigmented nodular epithelioid melanoma with mucosal ulceration invading bone (as shown in Figure 2). Lymphovascular invasion was present, and perineural invasion was absent; mitotic index 6–8/mm^2^. The tumour was present at inked/cauterized edges in fragmented specimens; margins were not assessable (Rx). Pathologic stage was pT4a Nx Mx (Melan-A/HMB-45 positive).

Early postoperative CT sinuses (8 April) showed extensive recurrent disease throughout the paranasal sinuses and nasopharynx with destruction of the left lamina papyracea and ethmoid roof erosion; there was no intracranial mass effect on CT. Brain MRI (9 April), compared with 10 March, demonstrated interval progression with cribriform plate/olfactory cleft destruction, dural thickening/infiltration in the anterior cranial fossa (no intra-axial enhancement), increasing left-orbital disease with extra-conal extension, extraocular muscle involvement, and optic nerve compression at the canal/apex, with more pronounced exophthalmos. The radiologic impression favoured T4b behaviour. Minimal mastoid/middle ear effusions and Fazekas 1 small-vessel changes were noted.

The tumour board (18 April) recommended palliative radiotherapy. Unfortunately, while waiting for therapy on 9 June, the patient was admitted emergently for nasal bleeding from a tumour, left exophthalmos, and severe cephalgia; haemostasis was achieved by endonasal electrocautery. Analgesia was escalated (tramadol, amitriptyline, carbamazepine, ketorolac, paracetamol; transdermal fentanyl increased from 25 to 50 μg/72 h). VMAT was delivered on 19 June–2 July (33 Gy in 11 fractions; the target was recorded in the chart as a maxillary-sinus field).

Neurological examination revealed limb ataxia, left total ophthalmoparesis with blindness OS, left peripheral facial paresis; CRP was 218 mg/L. During treatment, she developed severe right hip pain and weakness. Pelvic imaging (5–6 July) showed no lytic lesions or fractures; bilateral hip osteoarthrosis with right acetabular dysplasia and muscular fatty atrophy excluded metastatic fracture.

On 3 July, because of new central neurological signs (right-leg plegia), escalating pain, and clinical decline, radiotherapy was discontinued, and dehydration therapy commenced. She was transferred to palliative care (ECOG 4, total dependence). From mid-July to mid-August, she experienced recurrent bleeding from the tumour, severe nasopharyngeal/oral/back/hip pain, and progressive cachexia. The patient died on 14 August.

## 3. Discussion

It is known that SNMM carries a high risk of having a high local recurrence rate post-surgery [13]. This case illustrates exceptionally rapid local progression with skull-base and orbital involvement shortly after endoscopic piecemeal resection, when comparing diagnostic tests performed approximately 1 month apart, leading to rapid decline and progression of neurological symptoms, and the patient’s death 7 months after initial presentation.

Producing an apparent clinicoradiologic “up-staging” (pathologic pT4a vs. imaging features consistent with skull-base/orbital extension). Although MRI is central for preoperative assessment of dural and orbital invasion, systematic evidence across sinonasal malignancies indicates that false-negative findings can occur, and subtle early invasion (e.g., periorbital or dural involvement) may not be definitively excluded in every patient despite contrast-enhanced protocols [7]. In parallel, piecemeal endoscopic resection—sometimes unavoidable in anatomically constrained disease—can fragment specimens and limit reliable margin assessment, complicating postoperative risk stratification and highlighting the importance of structured specimen handling and mapping approaches where feasible.

Once skull-base invasion and vision-threatening orbital progression develop, management priorities can shift toward symptom control, hemorrhage/epistaxis management, ocular protection, and aggressive multimodal analgesia, integrated with timely goals-of-care discussions. Contemporary multidisciplinary frameworks for sinonasal malignancies emphasize individualized decision-making in locally advanced disease, where curative options may be limited, and morbidity from local progression can dominate the clinical course [6]. Regarding orbital management, contemporary aggregated evidence supports orbit-preserving approaches in appropriately selected sinonasal malignancies with orbital involvement, with overall survival comparable to orbit-sacrificing surgery and the potential to maintain a functionally useful eye; decisions should therefore be anchored in the degree of invasion, anticipated benefit, and patient-centred outcomes rather than default exenteration.

Radiotherapy remains a key modality for local control or palliation in mucosal melanoma of the head and neck, commonly improving locoregional control even when distant failure limits survival benefit [8]. Modern radiotherapy approaches and charged particle therapy have been increasingly discussed for sinonasal sites due to the proximity of critical organs; recent sinonasal mucosal melanoma experience with adjuvant proton therapy demonstrates feasibility and favourable early toxicity with encouraging early local control in small cohorts, supporting its consideration within multimodality pathways when available [8]. Nonetheless, systemic progression remains a major threat, and although immunotherapy and targeted therapies have expanded options, sinonasal mucosal melanoma continues to show heterogeneous responsiveness. Overall, this case supports early integration of structured palliation and symptom-directed protocols when rapid orbital compromise, refractory bleeding from tumour, and skull-base extension emerge, while maintaining multidisciplinary reassessment as the disease trajectory evolves.

## 4. Conclusions

Sinonasal mucosal epithelioid melanoma may demonstrate fulminant local progression with early skull-base and orbital involvement despite apparently limited preoperative imaging. When rapid vision loss, dural infiltration, and refractory nasal bleeding from the tumour develop, structured palliation, hemorrhage control, and aggressive multimodal analgesia become central to care and should be initiated early alongside ongoing multidisciplinary decision-making.

## Data Availability

Data is contained within the article.

## Abbreviations

The following abbreviations are used in this manuscript:

CSF	Cerebrospinal fluid
CT	Computed tomography
ECOG	Eastern Cooperative Oncology Group
FESS	Functional endoscopic sinus surgery
IHC	Immunohistochemistry
MRI	Magnetic resonance imaging
PET	Positron emission tomography
SNMM	Sinonasal mucosal melanoma
VMAT	Volumetric modulated arc therapy
